# Molecular Docking Optimization in the Context of Multi-Drug Resistant and Sensitive EGFR Mutants

**DOI:** 10.3390/molecules21111575

**Published:** 2016-11-19

**Authors:** María Jesús García-Godoy, Esteban López-Camacho, José García-Nieto, Antonio J. Nebro, José F. Aldana-Montes

**Affiliations:** Khaos Research Group, Departament of Computer Sciences, University of Málaga (UMA), ETSI Informática, Campus de Teatinos, 29071 Málaga, Spain; mjgarciag@lcc.uma.es (M.J.G.-G.); esteban@lcc.uma.es (E.L.-C.); jnieto@lcc.uma.es (J.G.-N.); antonio@lcc.uma.es (A.J.N.)

**Keywords:** molecular docking, metaheuristics, multi-objective optimization, drug resistance, epidermal growth factor, Epidermal Growth Factor Receptor, Epidermal Growth Factor Receptor mutants

## Abstract

The human Epidermal Growth Factor (EGFR) plays an important role in signaling pathways, such as cell proliferation and migration. Mutations like G719S, L858R, T790M, G719S/T790M or T790M/L858R can alter its conformation, and, therefore, drug responses from lung cancer patients. In this context, candidate drugs are being tested and in silico studies are necessary to know how these mutations affect the ligand binding site. This problem can be tackled by using a multi-objective approach applied to the molecular docking problem. According to the literature, few studies are related to the application of multi-objective approaches by minimizing two or more objectives in drug discovery. In this study, we have used four algorithms (NSGA-II, GDE3, SMPSO and MOEA/D) to minimize two objectives: the ligand–receptor intermolecular energy and the RMSD score. We have prepared a set of instances that includes the wild-type EGFR kinase domain and the same receptor with somatic mutations, and then we assessed the performance of the algorithms by applying a quality indicator to evaluate the convergence and diversity of the reference fronts. The MOEA/D algorithm yields the best solutions to these docking problems. The obtained solutions were analyzed, showing promising results to predict candidate EGFR inhibitors by using this multi-objective approach.

## 1. Introduction

The human Epidermal Growth Factor (EGFR) has an important role in multiple signaling pathways such as cell proliferation and migration. The epidermal growth factor (ligand) binds to the extracellular EGFR domains, activating the tyrosine kinase domain. Mutations in the EGFR kinase domain cause non-small cell lung cancer. Mutations such as G719S, L858R, T790M, G719S/T790M or T790M/L858R can alter the kinase activity of EGFR by disrupting autoinhibitory interactions [1], which are associated with different drug responses from non-small cell lung cancer patients. In this context, new treatments are being tested on patients that have these mutations in EGFR. In order to understand the mechanisms of how these mutations affect the drug binding site, in silico studies like molecular docking are applied in these cases.

In this study, our motivation is to experiment with molecular instances whose receptors are multi-drug resistant and sensitive EGFR mutants, in order to test the accuracy of the ligand’s conformation prediction in docking simulations. With this purpose, we took a multi-objective approach consisting of minimizing two objectives: the root-mean-square deviation and the intermolecular energy.

In [2], the authors indicated that, in non-small cell lung cancer patients, it can be very beneficial to take biopsies and analyze the EGFR mutations to select appropriate treatments with new candidate drugs. This requires the application of accurate molecular docking approaches, especially multi-objective strategies that minimize more than one objective, and, therefore, the practitioners have a range of solutions to select.

In this sense, the use of global optimization algorithms to tackle with the molecular docking problem has been extensively studied in the past with successful results when using single-objective [3] and multi-objective methods [4,5,6,7,8]. In the case of multi-objective optimization, a series of proposals have been appearing since 2015, which involve flexibility in the side-chains of the receptor’s active site and the use of the energy scoring function provided by AutoDock (version 4.2 , The Scripps Research Institute, California, EEUU), which is one of the most used and cited tools for drug discovery. García-Godoy et al. [9] studied a set of representative multi-objective algorithms, namely: two variants of NSGA-II, the third evolution step of generalized differential evolution (GDE3), the multi-objective evolutionary algorithm on decomposition (MOEA/D) and the S-metric evolutionary multi-objective optimization (SMS-EMOA). The objectives to optimize were the intermolecular and intramolecular energies. The set of molecular instances used for this study involved 11 compounds of ligands with different sizes and flexible residues in the active sites of the HIV-proteases. In addition, two use cases based on drug discovery with the EGFR and the aeroplysinin-1 (an antiangiogenic compound) were analyzed to show the proper application of this multi-objective approach. In 2016, López-Camacho et al. [10] proposed minimizing two different objectives from the AutoDock energy function: the intermolecular energy and the RMSD (Root Mean Square Deviation) as two objectives to evaluate the quality of the ligand–protein interactions. In this last work, the molecular compounds used based on HIV-proteases and retroviral inhibitors [11]. The results demonstrated that the SMPSO showed the best overall results in terms of binding energy and RMSD scores (values lower than 2 Å) compared to those obtained by the rest of algorithms. These results are useful in those cases in which the crystallographic structure exists, and, therefore, the ligand conformation to the receptor is known.

Following the research line of these last studies, in this work, we are driven by the motivation of evaluating our multi-objective approach by testing it in the context of instances whose receptors are multi-drug resistant and sensitive. We aim at checking our proposed optimizers in changing environments guided by mutations in receptors. The contributions of this paper can be summarized as follows:

- In the study performed in [9], the two objectives to minimize were the intermolecular and intramolecular energies. The first objective represents the difference between the unbound and bound states of the receptor and ligand, and the second one represents the unbound and bound states of the ligand-receptor complex. In this paper, our problem has a different bi-objective formulation, being the objectives to minimize the RMSD score and the intermolecular energy. The RMSD score is a similarity measure to determine the quality of the results obtained by the docking simulations when the co-crystallized ligand is available.
- In [10], we carried a comparative study of a number of algorithms when solving the benchmark based on HIV-proteases proposed by Morris et al. [11]. In this paper, we have focused on the prediction of ligands’ conformations to the EGFR, whose structure can be altered by mutations in lung cancer patients. The results of the two papers are different: in [10], the best performant algorithm was SMPSO, while, in current paper, the most salient algorithm is MOEA/D.
- We have selected a set of instances whose receptors are wild-type or contain the mutations T790M/G719S, G719S, L858R, T790M and T790M7/L858R. The tyrosine kinase inhibitors present different numbers of active torsions. The obtained results were selected according to the contribution of the intermolecular energy and the RMSD score. In addition, the docking solutions were analyzed considering the ligand’s binding site and the molecular interactions. To do that, we have classified the docking solutions into different groups according to the somatic mutations presented in the EGFR receptor. For each group, we have included the atoms implied in the predicted H-bonds. The predicted ligand’s conformations have been also compared with the co-crystallized ligands’ structures.

The remaining of this article is organized as follows. Section 2 briefly describes the multi-objective approach used for molecular docking optimization. In Section 3, the experimental framework and the molecular compounds generated to carry out this study are detailed. Section 4 is devoted to report results and analysis focusing on binding sites in EGFR and molecular interactions. Finally, Section 5 outlines concluding remarks and future work.

## 2. The Multi-Objective Docking Strategy

A multi-objective problem consists of two spaces: the decision and the objective spaces. The decision space involves all the possible solutions. The objective space includes the objective values. A complete definition of a multi-objective problem based on the decision space can be found in [9]. In the case of the objective space, we have treated it as a multi-objective approach. In this paper, the objectives to optimize are the intermolecular energy, which is the sum of the bound and unbound states of the ligand and receptor and the ligand-receptor complex and the RMSD score. The intermolecular energy is defined as follows:

(1) $$E_{binding}=Q_{bound}^{R-L}+Q_{unbound}^{R-L}$$

The RMSD is a measure of similarity between the real ligand position in the receptor and the predicted position of the docking ligand. The lower the RMSD score, the better the docking solution is. The RMSD cutoff of 2 Å is widely considered as a criterion to consider the computed ligand–protein conformation as a good prediction [12]. The RMSD score for two identical structures *a* and *b* is defined as follows:

(2) $${RMSD}_{ab}=max\left({RMSD}_{ab}^{\prime },{RMSD}_{ba}^{\prime }\right),\;with\;{RMSD}_{ab}^{{}^{\prime }}=\sqrt[{}]{\frac{1}{N}\sum_{i}\underset{j}{min}\;r_{2}^{ij}}$$

The sum is over all *N* heavy atoms in structure *a*, the minimum is over all atoms in structure *a*, with the same element type as atom *i* in structure *b*.

## 3. Experiments Section

In this study, four algorithms which are representative of the state-of-the-art in the multi-objective optimization field were selected: NSGA-II [13], GDE3 [14], SMPSO [15] and MOEA/D [16]. These algorithms were also included in [10] to solve the proposed benchmark based on HIV-proteases and inhibitors with different sizes. As mentioned in Section 1, the results obtained showed that SMPSO and MOEA/D returned the best and the second best solutions, especially in those complex cases where the inhibitor has a small size, and, therefore, the conformational search space increases. Some studies indicated that LGA fails in finding an accurate ligand’s conformation to these cases [11]. These promising results have led us to apply this multi-objective approach to solve real problems in which accurate docking techniques are necessary in in silico studies.

When comparing the performance of different single-objective optimization algorithms, only the best obtained value by each algorithm (in the case of minimization, the lowest value) needs to be observed. However, this is not possible when considering multi-objective optimization problems into consideration as a set of ‘best’ values are obtained. In this case, an approximation set to the optimal Pareto front of the problem is computed, to be compared with the obtained set of solutions. The properties that are usually required are convergence and a uniform diversity. In this paper, we have chosen the Hypervolume ($I_{HV}$) quality indicator, as it validates both convergence and diversity.

The instances selected correspond to 35 protein-ligand complexes with flexible ligands. The crystallographic structures of these instances have been taken from the PDB (Protein Data Bank) database [17]. Table 1 summarizes the set of problems showing the name of the ligand and receptor entities, the mutation that affects the EGFR receptor, the PDB accession code, the resolution of the crystallographic structure (Å), and the number of active torsions of the ligand. In terms of flexiblity, for all the ligands of the instances, the maximum number of active torsions allowed were applied, selecting those torsions that permit the fewest number of atoms to move around the ligand’s core.

This set of instances includes the wild-type EGFR kinase domain and EGFR kinase domain with mutations, which are involved in types of cancer like non-small cell lung cancer [1]. These mutations in the EGFR kinase domain have been studied intensely to discover the mechanisms of activation and effects on the ligand binding. For example, the G719S mutation is located in the N-terminal lobe of the kinase domain of EGFR, within the P-loop. Some studies in docking and dynamic simulations have shown that the mutation G719S causes ligands to move closer to the hinge region of EGFR, which connects the N-terminal and C-terminal lobes of the domain kinase [18]. This mutation is associated with increased sensitivity to the EGFR TKIs (tyrosine kinase inhibitors), erlotinib (Tarceva) and gefitinib (Iressa) [19]. The mutation T790M allows the ligand to escape from the binding pocket according to studies based on binding pocket. This mutation causes that more than 50% of EGFR-mutated lung cancers develop acquired resistance to erlotinib or gefitinib [20]. The double mutation T790M/G719S like the mutation T790M alters the ligand-EGFR kinase domain binding and allows the ligand to escape. This mutation has no individual effects as mutations, but rather that the mutation T719M reverses the effect of the mutation G719S [18]. The mutation L858R makes the EGFR kinase domain more sensitive to erlotinib and gefitinib than the wild-type, and, therefore, increases the survival rate of patients that undergo TKI treatment [21]. Finally, a recent study reported that the double mutant L858R/T790M in EGFR domain kinase had a poor response to gefitinib in patients with lung adenocarcinoma [2].

The AutoDock 4.2 and jMetalCpp (version 1.7 , Khaos group, Málaga, Spain) were used to perform the docking experiments [22]. The jMetalCpp framework provides the multi-objective algorithms and AutoDock 4.2 evaluates the generated solutions according to its energy function. Before the execution of each algorithm, we prepared the ligand and macromolecule. Both structures were separated by Chimera UCSF software (version 1.8, UCSF, San Francisco, California, EEUU) [23] and saved as PDB files. These files were processed by removing non-interacting ions, solvent molecules, etc. Several Python scripts were implemented to generate the PDBQT (Protein Data Bank with partial charges and atom type) non-interacting ions, solvent molecules of the ligands and macromolecules. The Python scripts were prepare_ligand4.py and prepare_receptor4.py in which the parameters were configured. For the ligands, the number of active torsions were the maximum according to the ligand’s conformation. For the receptors, the non-polar hydrogens were merged. Gasteiger charges were added to the ligands and the receptors. The grid maps were calculated using AutoGrid (version 4.2, The Scripps Research Institute, California, EEUU) with the coordinates 60 Å × 60 Å × 60 Å and a grid spacing of 0.375 Å was set. A docking parameter file was also created as an input to AutoDock 4.2 and jMetalCpp in which 31 independent runs were defined. From the results of these experiments, we have calculated the median and interquartile range (IQR) as measures of location (or central tendency) and statistical dispersion, respectively.

## 4. Results and Discussion

In this section, we have carried out a comparative analysis of the results returned by the SMPSO, GDE3, MOEA/D and NSGA-II algorithms. In addition, an analysis based on ligand binding sites and molecular interaction have been reported.

### 4.1. Algorithm Comparative Analysis

In Table S1, the medians and the interquartile ranges are shown for the $I_{HV}$ quality indicator for the set of 35 molecular instances. These instances are classified in seven groups according to whether the EGFR is the wild type or is mutated. For the $I_{HV}$, the higher the median value, the better the result. As shown in Table S1, the best overall results were obtained by MOEA/D (best medians and second best medians with dark gray and light gray backgrounds, respectively). For the instances 2j6m, 2itp, 2itz, and 5em7, the best median values were obtained by GDE3. For the instances 2ito and 4rj5, the best median values for $I_{HV}$ were obtained by NSGA II. We have applied the hypervolume, which is a quality indicator that quantifies and encapsulates the convergence and the diversity with respect to the Pareto front approximations. It is worth noting that molecular docking is a real optimization problem so the optimal Pareto front is not known. To address this issue, we have generated reference fonts with all the non-dominated solutions obtained for each instance from the executions of all the algorithms. Therefore, according to the previous report, MOEA/D has the best results in 27 out of the 35 instances and the second best results in seven instances; while GDE3 obtains the best results in six out of the 35 instances and the second best in 17 instances.

To present the results obtained with statistical confidence, we have carried out a series of non-parametric statistical tests applying a confidence of α = 0.05. To do so, Friedman’s ranking and Holm’s post hoc multi-comparison tests have been applied to know which algorithms are statistically worse compared to the control one, which, in this case, is MOEA/D (the algorithm with the best ranking).

As shown in Table 2, MOEA/D reaches the best ranking value according to Friedman’s rank for $I_{HV}$ with 1.24, followed by GDE3 with 2.30, NSGA-II with 3.11 and SMPSO with 3.34. In line with these results, MOEA/D was established as the control algorithm to be used in Holm’s post hoc multi-comparison test to be compared with the remaining algorithms. The Holm’s adjusted *p*-values in Table 2 show lower values than the confidence level. This indicates that MOEA/D is statistically better than GDE3, NSGA-II and SMPSO, for the molecular instances used here.

**Figure 1:**
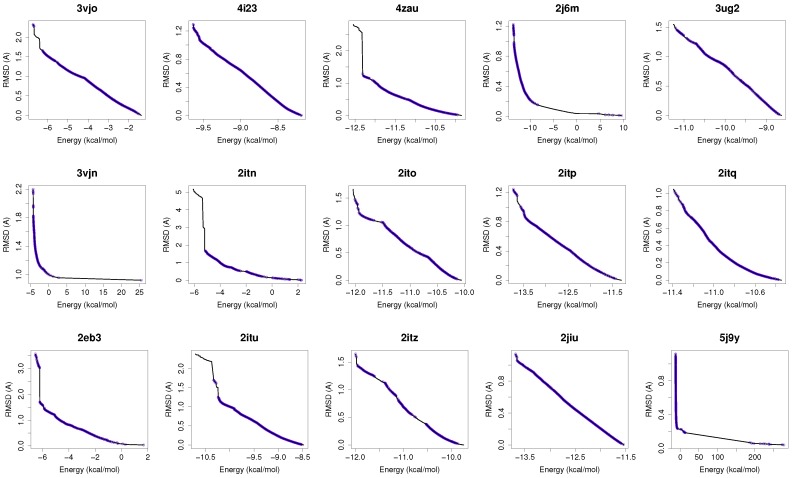

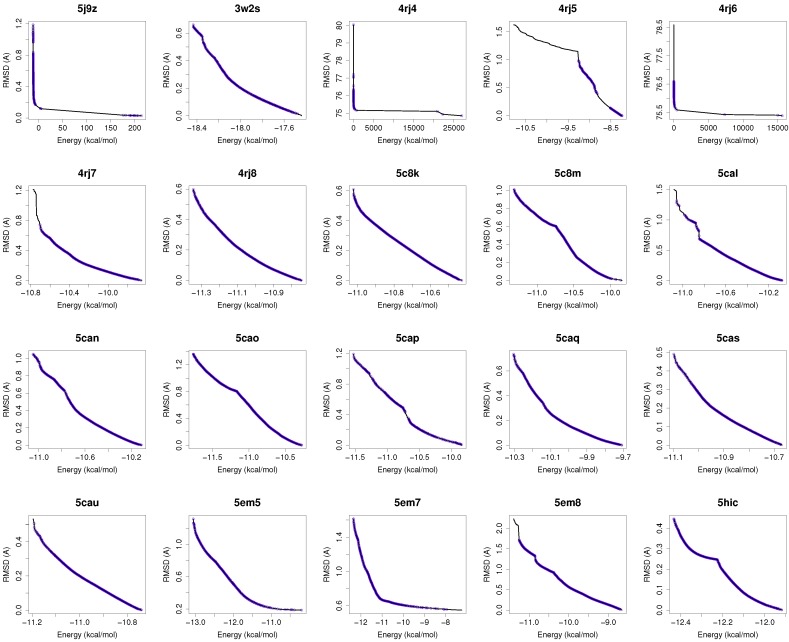
MOEA/D contributions to the reference fronts of all the molecular instances. In each plot, the reference front is represented with a solid **black** line. The front of the non-dominated solutions are shown with a **blue** line.

Figure 1 shows the reference fronts (solid line) and the resulting Pareto fronts of MOEA/D with the non-dominated solutions (blue line). As shown, MOEA/D contributes to the reference fronts with the greatest amount of obtained solutions in most of the analyzed instances (e.g., 4i23, 2itz, 2jiu, etc.). This figure is consistent with the statistical results that are shown in Table 2. In this figure, it can be easily observable that in those instances where the algorithm found lower RMSD values (ligand’s conformations closer to the co-crystallized ligand) and lower intermolecular energies of the ligand-receptor complexes, MOEA/D achieves the best results. However, there are fonts in which MOEA/D does not contribute to solutions that have a lower ligand-receptor intermolecular energy and higher values of RMSD. These cases are presented in the instances 2itn, 2itu and 4rj5. In these cases, NSGA-II obtained solutions with lower values of intermolecular energy and higher values of RMSD. GDE3, despite being the second best ranked algorithm, obtained all of its solutions in the same region of the reference front as MOEA/D, so they show similar performances with overlapping behaviors.

### 4.2. Analysis on Binding Sites in EGFR and Molecular Interactions

This subsection is devoted to examine selected uses cases based on the wild-type EGFR, EGFR with mutation G719S, EGFR with mutation L858R, and EGFR with double mutations T790M/L858R.

#### 4.2.1. Molecular Docking Analysis with the Wild-Type EGFR

For a use case based on the wild-type EGFR, we have chosen the instance 4zau, as it shows a reference front in Figure 1 with diverse solutions with RMSD values in the range of (0.02–9.9) Å and the intermolecular energies from −9.9 kcal/mol to −12.3 kcal/mol.

**Figure 2:**
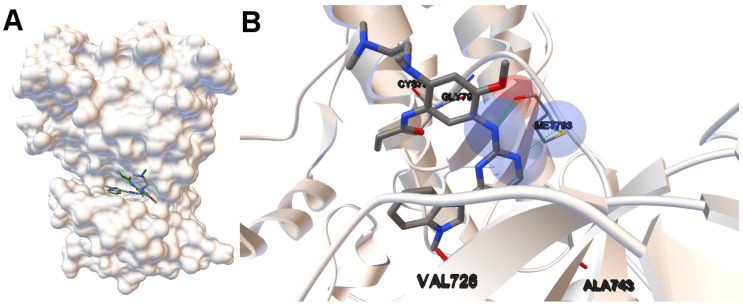
(**A**) the tri-dimensional structure of the human wild-type EGFR kinase domain for the instance 4zau in which the cleft can be easily observable. The molecular surface is represented using ADTools (version 1.5.6, The Scripps Research Institute, California, EEUU). The computed (in **green**) and the co-crystallized ligands are also represented. The low value of the **RMSD** of the computed ligand returned by the algorithm MOEA/D does not allow the two ligands to be represented separately given that they overlap; (**B**) the molecular interactions between the ligand AZD9291 and the EGFR domain kinase. The H-bonds are represented by **green** spheres. The atoms involved in these H-bonds are represented by colored spheres according to the atom type. In this case, the nitrogen and oxygen atoms are represented in **blue** and **red** spheres, respectively. The aminoacids closer to the interactions and the aminoacid(s) involved in the H-bond(s) are also labeled.

EGFR consists of a single transmembrane domain, extracellular and intracellular kinase domain. Image (A) in Figure 2 shows the 3D structure of the human wild-type EGFR domain kinase of the complex 4zau. The kinase domain of EGFR consists of an N-terminal lobe, C-terminal lobe and a hinge region which connects these two lobes. The active site is located in the cleft formed by these two lobes. The 4zau crystallographic structure contains the AZD9291 transferase inhibitor and the EGFR domain kinase that includes residues 696 to 1022 (a total length of 330 residues). In image (A), the reference (co-crystallized) and the computed ligand AZD9291 are represented. Given the RMSD score of the ligand returned by the MOEA/D equals to 0.03 Å, the representation of the reference and computed ligands to the EGFR domain kinase is overlapped. The ligand-receptor intermolecular energy of the computed ligand corresponds to −8.77 kcal/mol and the final free binding energy to −6.09 kcal/mol (with the torsional free energy added to the sum of energies). Image (B) represents the molecular interactions between the computed ligand AZD9291 and the EGFR domain kinase. The H-bonds are represented by green spheres and the atoms involved in this interaction are represented by colored spheres (the colour of each sphere depends on the atom type). AZD9291 N3 hydrogen bonds to the hinge atom MET793 amide N through a H-bond. AZD9291 N4 bonds to the MET793 amide -NH through a H-bond. This predicted conformation of AZD9291 is in accordance with the ligand’s conformation reported in [24].

**Figure 3:**
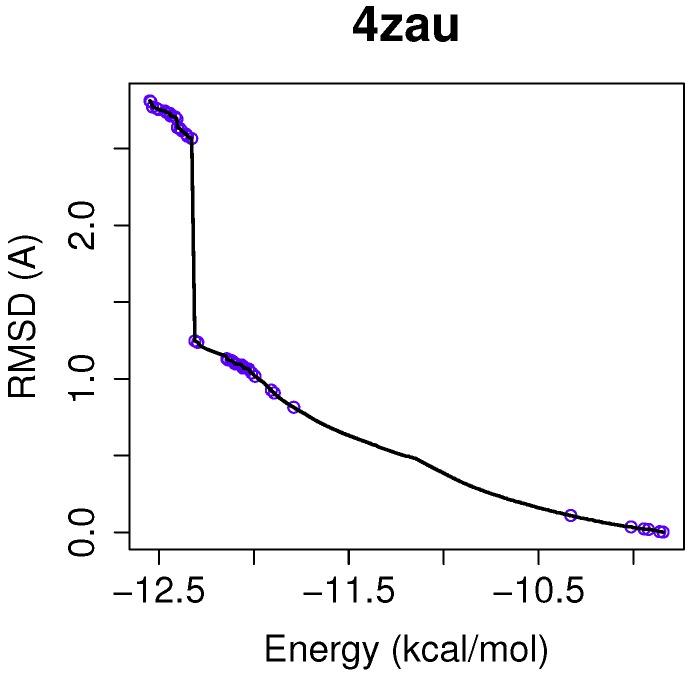
The reference front and the non-dominated solutions are represented in **black** and **blue**, respectively. It can be observed that the non-dominated solutions returned by NSGA-II present diversity in the reference front in which the intermolecular energy values (kcal/mol) are more negative and the RMSD scores are higher.

In this example of the human wild-type EGFR domain kinase and the ligand AZD9291, we have chosen one of the results returned by the algorithm NSGA-II from the set of non-dominated solutions, which shows a high diversity in the reference front in cases when the ligand-receptor intermolecular energy values are lower, and the RMSD scores show higher values. This is shown in Figure 3, in which the reference front is represented by a solid black line, and the non-dominated solutions returned by NSGA-II for the instance 4zau are represented in blue.

**Figure 4:**
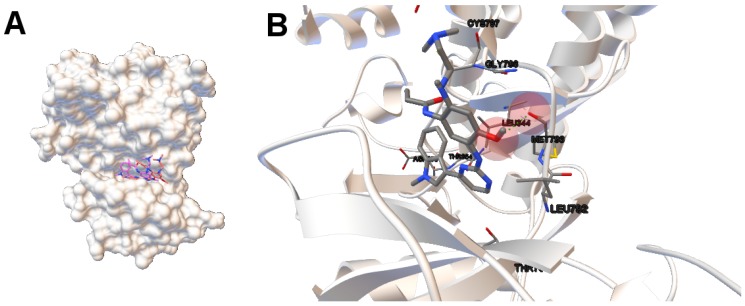
(**A**) the tri-dimensional structure of the human wild-type EGFR domain kinase. The molecular surface is represented using ADTools. The computed and reference ligands are represented in **pink** and **red**, respectively. Both ligands’ conformations are bound on the outer edge of the cleft of the ATP-binding; image (**B**) shows the molecular interactions between the computed conformation of the ligand AZD9291 and the EGFR domain kinase (its secondary structure is also shown). The atoms involved in the H-bonds are represented by colored spheres. The H-bond is represented by **green** spheres.

Image (A) in Figure 4 shows the tri-dimensional representation of a non-dominated solution from the NSGA-II. In this case, the solution has a lower intermolecular energy (−10.67 kcal/mol) and an RMSD score higher (2.81 Å ) than the solutions presented above. The reference ligand is shown in red and the computed ligand in pink. As shown in image (A), the computed and reference ligands are not overlapped as in the last example. It is remarkable that although the solution has a higher RMSD score, the computed ligand binds on the outer edge of the ATP-binding pocket of the EGFR domain kinase as in the previous example. In image (B), the molecular interactions are shown. The ligand AZD9291 and the MET793 amide oxygen interacts through a H-bond. The involved atoms are shown as red spheres (Oxygen atoms). The H-bond is represented with green spheres. The computed ligand’s conformations returned by MOEA/D and NSGA-II are in agreed with those results presented by Yosaatmadja et al., 2015 [24], which lead us to suggest that our approach is able to detect real conformations obtained from past crystallographic studies.

#### 4.2.2. Molecular Docking Analysis on the EGFR That Contains the Mutations G719S and L858R

The mutation G719S is less frequent than other mutations like the L858R [25]. This mutation affects the phosphate-binding loop (P-loop) of the EGFR domain kinase. This mutation replaces the GLY719 with SER. The cells that show this mutation display oncogenic properties, but they are more sensitive to TK inhibitors than the wild-type kinase domain that does not contain this mutation [26]. The L858R mutation is more frequent than other somatic mutations in EGFR. This mutation replaces LEU858 with ARG. This mutation makes EGFR more sensitive to TK inhibitors than the wild-type EGFR [25].

**Figure 5:**
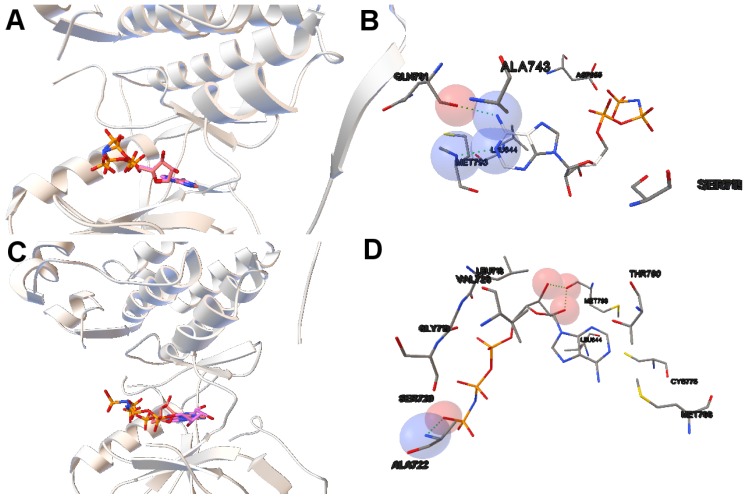
(**A**,**C**) show the tri-dimensional kinase domains of the instances 2itn and 2eb3 with the mutations G719S and L858R, respectively. The computed and the reference (AMP-PNP) ligands are represented in **red** and **pink**, respectively. The computed ligands adopt the conformation of the reference ligands; (**B**,**D**) show the molecular interactions between the computed ligands AMP-PNP and the kinase domains of EGFR. The atoms involved in the H-bonds are shown as spheres. The color of these depends on the atom type. The H-bonds are represented by **green** spheres. The residues are also labeled, including the residues closer to the ligand–macromolecule interaction.

In this study, we have chosen two instances: the 2itn and 2eb3. The instance 2itn corresponds to the crystal structure of EGFR kinase domain G719S mutation in complex with AMP-PNP. As shown in Figure 1, the non-dominated solutions returned by MOEA/D for the instance 2itn contribute to those solutions with values of lower RMSD and minimized intermolecular energies. In this region, we have chosen a non-dominated solution whose ligand-receptor intermolecular energy equals −3.03 kcal/mol and the RMSD value is 0.18 Å. The instance 2eb3 corresponds to the crystal structure of mutated EGFR kinase domain (L858R) in a complex with AMP-PNP. A non-dominated solution was chosen from the set of non-dominated solutions (Figure 5) with an RMSD value of 3.5 Å and an intermolecular energy of −6.53 kcal/mol. In Figure 5, images A and C show that the computed ligand position is very similar to the reference ligand. Image B shows that the N1 and N6 of AMP-PNP forms two H-bonds with the the -NH of MET793 amide and the GLY791 amide oxygen, respectively. Image D represents an H-bond formed between O2 and O3 of AMP-PNP with the MET793 amide nitrogen. In both instances, the residue MET793 is involved in the receptor-ligand interactions. These results are the same as those reported by Yun et al. [27] that showed that structures’ conformation of either TK inhibitors or AMP-PNP in crystallized structures of EGFR with these two mutations (and also wild-type EGFR) are very similar as shown in Images A and C in Figure 5.

#### 4.2.3. Molecular Docking Analysis on the EGFR Double Mutants T790M/L858R and T790M/G719S

As mentioned before, patients with non-small cell lung cancer have exhibited mutations. The majority of those patients with EGFR mutations have benefited from taking TK inhibitors. These EGFR mutations like G719S ad L858R make the receptor more sensitive to TK inhibitors such as gefitinib and erlotinib. However, double mutations like T790M/G719S and T790M/L858R have been reported to create acquired resistance to TK inhibitors in patients with lung cancer [2,18]. For example, in a recent study [2], a patient with lung adenocarcinoma did not respond to TK inhibitors. Genetic studies showed that this patient had the EGFR double mutant T790M/L858R, which alters the response to these inhibitors. Another interesting oncogenic double mutation in EGFR is T790M/G719S. This mutation T790M reverts the effect of mutation G719S that results in patients with this double mutation not responding to the TK inhibitors’ treatment. In this context, there are studies [25] that have demonstrated acquired resistance of this double mutant to TK inhibitors like gefitinib and other analyses using docking and dynamics simulations that show the change of structure of this double mutation and how other ATP-competitive inhibitors can be designed according to the structure of this double mutant.

In this study, we have applied the multi-objective approach to a set of instances in which EGFR contain the double mutations T790M/L858R and T790M/G719S as shown in Table 1. For the analysis of the results obtained from the docking simulations, we have chosen the instances 4rj5 and 3ug2. For 4rj5, we have chosen a non-dominated solution returned by MOEA/D from the set of solutions with low RMSD values and intermolecular energies from −8.5 to −9.0 kcal/mol. The solution presents an RMSD value of 0.001 Å and an intermolecular energy value of −8.24 kcal/mol.

**Figure 6:**
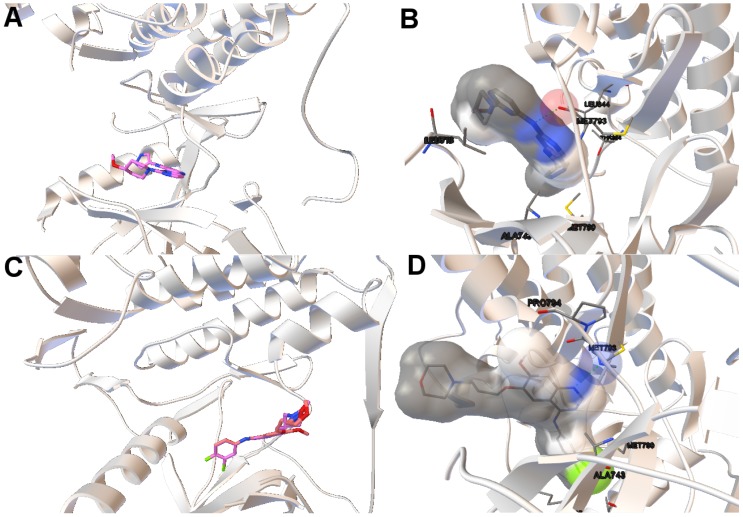
(**A**,**C**) show the tri-dimensional structure with the reference/computed ligands for 4rj5 and 3ug2, respectively. The reference and computed ligands are represented in **red** and **pink**, respectively; images (**B**,**D**) show the molecular interaction between ligands and EGFR double mutant. The atoms involved in H-bonds are represented by colored spheres. H-bonds are represented by **green** spheres. The residues are also labeled, including the residues closer to the ligand–macromolecule interaction.

Image A in Figure 6 shows the reference and computed ligand represented. Both ligands’ conformations overlap because of the low RMSD score of the ligand’s conformation returned by MOEA/D. Image B in Figure shows a H-bond represented by green spheres that is formed by MET793 amide oxygen and N15 of the diaminopyrimidine-based inhibitor (compound 5 with ID:3QY in PDB database). For 3ug2, as the diversity of MOEA/D is high (Figure 1), we have selected a non-dominated solution from the extreme of the set of non-dominated solutions that show a high diversity when the gefitinib-EGFR double mutant complexes are more stable in terms of intermolecular energy and values of RMSD are lower than 2Å. Image C in Figure 6 shows the overlapped reference and computed gefitinib. Image D shows the computed gefitinib to the EGFR double mutant. An H-bond is formed between the N3 of gefitinib and the -NH of MET793 amide.

## 5. Conclusions

In this study, we perform a through validation of a series of multi-objective optimization algorithms for molecular docking in the context of a set of mutant instances focused on the kinase domain of EGFR. In this approach, the inhibitors’ conformation are predicted in terms of energy and RMSD (compared with its corresponding co-crystallized ligand). The EGFR domain kinase contains the mutations G719S, L858R, T790M, G719S/T790M or T790M/L858R, which can alter the kinase activity of EGFR by disrupting autoinhibitory interactions [1]. The ligands of the selected complexes are small-molecule kinase inhibitors. Our goal is to check that these mutations in the EGFR kinase domain have also displayed different binding modes of kinase inhibitors for the EGFR kinase domain. This has been previously suggested in several studies, which could lead to differences in the inhibitor sensitivity of these targets, and, therefore, different responses from patients with non-small lung cancer undergoing treatment with these drugs.

A series of conclusions can be extracted from both algorithmic and biological points of view, as follows:

- In [10], we have performed a preliminary algorithm comparative using the benchmark proposed by Morris et al [11]. In this study, we have applied a set of algorithms to solve real problems based on candidate drugs to be tested in non-small cell lung cancer patients with different drug responses.
- The minimization of the intermolecular and intramolecular energies to find ligands’ conformations to multi-drug and sensitive EGFR mutants resulted in low RMSD scores and intermolecular energy values in most of the analyzed instances. In general, MOEA/D shows the best numerical results, as it obtains lower RMSDs and intermolecular energy values than NSGA-II, GDE3, and SMPSO. However, there are some instances (2itn, 2itu and 4rj5) for which NSGA-II obtains lower values of intermolecular energies. This may be explained by the existence of different active sites in these molecular instances.
- For most of studied molecular instances, MOEA/D converges to the region biased towards minimum RMSD, whereas NSGA-II generates its fronts of non-dominated solutions in a different region, thereby giving cue to energy optimization.
- We have performed an analysis on binding sites in the EGFR kinase domain and molecular interactions. The use cases are based on instances with wild-type EGFR, EGFR with mutations L858R and G719S and EGFR double mutants (T790M/L858R and T790M/G719S). In the first group, the predicted ligand’s conformations of the instance 4zau are in accordance with the crystallographic studies as shown in [25]. In the second, the predicted ligands’ conformation for the instances 2itn and 2eb3 present similar ligands’ conformations to EGFR as those reported by [27]. In the third, the instances 4rj5 and 3ug2 contain EGFR double mutants. Patients diagnosed with lung cancer harboring these EGFR double mutation do not respond to EGFR-TKI. In these cases, it would be very beneficial to detect these EGFR mutations for selecting appropriated treatments and do previous docking simulations with accurate techniques like those proposed in this paper.
- The proposed approach can be used for in silico studies to test other analog kinase inhibitors or similar compounds for drug discovery in those cancers in which therapeutic targets are changed by somatic mutations.

As ongoing and future work, we are designing hybrid algorithms combining search procedures from both MOEA/D and NSGA-II in order to get solutions covering the full Pareto front. In this regard, the use of local search and restarting methods could be beneficial for such experiments in which other alternative active sites in compounds are required to be explored and discovered.

## Figures and Tables

**Table 1:** X-ray crystal structure coordinates taken from PDB database. Their accession codes from the PDB database, type of the receptor’s mutation, resolution in Å, and number of active torsion added to the ligands are presented.

Protein–Ligand Complexes	Type of Mutation	PDB Code	Resolution (Å)	No. of Active Torsions
EGFR/AMPPNP	Wild type	3vjo	2.64	8
EGFR/Dacomitinib	Wild type	4i23	2.8	3
EGFR/AZD9291	Wild type	4zau	2.8	9
EGFR/AEE788 inhibitor	Wild type	2j6m	3.1	7
EGFR/Gefitinib	T790M/G719S	3ug2	2.5	8
EGFR/AMP-PNP	T790M/G719S	3vjn	2.34	8
EGFR/AMP-PNP	G719S	2itn	2.47	8
EGFR/Iressa	G719S	2ito	3.25	8
EGFR/AEE788	G719S	2itp	2.74	7
EGFR/AFN941	G719S	2itq	2.68	6
EGFR/AMP-PNP	L858R	2eb3	2.84	8
EGFR/AFN941	L858R	2itu	2.8	6
EGFR/Iressa	L858R	2itz	2.8	8
EGFR/AEE788	T790M	2jiu	3.05	7
EGFR/Pyrazolopyrimidine	T790M	5j9y	2.8	3
EGFR/Pyrazolopyrimidine inhibitor a1	T790M	5j9z	2.5	3
EGFR/Inhibitor compound **4**	T790M/L858R	3w2s	1.9	11
EGFR/Inhibitor compound **6**	T790M/L858R	4rj4	2.78	5
EGFR/Inhibitor compound **5**	T790M/L858R	4rj5	3.1	4
EGFR/Inhibitor compound **4**	T790M/L858R	4rj6	2.7	4
EGFR/Inhibitor compound **1**	T790M/L858R	4rj7	2.55	6
EGFR/Inhibitor compound **8**	T790M/L858R	4rj8	2.5	4
EGFR/Inhibitor compound **17**	T790M/L858R	5c8m	2.9	4
EGFR/Inhibitor compound **1**	T790M/L858R	5c8k	3	4
EGFR/Inhibitor compound **24**	T790M/L858R	5cal	2.7	6
EGFR/Inhibitor compound **27**	T790M/L858R	5can	2.8	4
EGFR/Inhibitor compound **29**	T790M/L858R	5cao	2.6	6
EGFR/Inhibitor compound **30**	T790M/L858R	5cap	2.4	7
EGFR/Inhibitor compound **33**	T790M/L858R	5caq	2.5	4
EGFR/Inhibitor compound **41a**	T790M/L858R	5cas	2.1	6
EGFR/Inhibitor compound **41b**	T790M/L858R	5cau	2.25	6
EGFR/pyridone compound **2**	T790M/L858R	5em5	2.65	7
EGFR/pyridone compound **13**	T790M/L858R	5em7	2.81	12
EGFR/pyridone compound **13**	T790M/L858R	5em8	2.8	6
EGFR/pyridone compound **13**	T790M/L858R	5hic	2.6	6

**Table 2:** Average Friedman’s rankings with Holm’s Adjusted *p*-values (0.05) of compared algorithms for the test set of 35 docking instances. Symbol * indicates the control algorithm which, in this case, is MOEA/D.

Hypervolume ($I_{\mathrm{HV}}$)		
Algorithm	Friedman’s Rank	Holm’s Adjusted *p*-Value
MOEA/D *	**1.24**	-
GDE3	2.30	6.13 × 10${}^{-4}$
NSGA-II	3.11	2.65 × 10${}^{-9}$
SMPSO	3.34	3.03 × 10${}^{-11}$

## Supplementary Materials

Supplementary materials can be accessed at: http://www.mdpi.com/1420-3049/21/11/1575/s1.
