# Supplementary material for: Molecular Docking Optimization in the Context of Multi-Drug Resistant and Sensitive EGFR Mutants
Source: Molecules. 2016 Nov 19;21(11):1575. doi: 10.3390/molecules21111575 (PMC6274512; doi:10.3390/molecules21111575)
Supplement: Supplementary file 1 [file molecules-21-01575-s001.pdf]

Supplementary Materials: Molecular Docking Optimization in the Context of Multi-Drug Resistant and Sensitive EGFR Mutants

María Jesús García-Godoy, Esteban López-Camacho, José García-Nieto, Antonio J. Nebro and José F. Aldana-Montes

Table S1. Median and Interquartile range of I_{HV} quality indicator for each algorithm (SMPSO, GDE3, MOEA/D and NSGAII) and instance. The best and second best median values are shown in dark gray and light gray backgrounds, respectively.

	SMPSO	GDE3	MOEA/D	NSGA-II
Wild type				
3vjo	$4.57 \times 10^{-1}_{3.0 \times 10^{-2}}$	$4.16 \times 10^{-1}_{8.3 \times 10^{-2}}$	$5.12 \times 10^{-1}_{9.3 \times 10^{-2}}$	$3.75 \times 10^{-1}_{1.1 \times 10^{-1}}$
4i23	$5.48 \times 10^{-1}_{8.0 \times 10^{-3}}$	$5.63 \times 10^{-1}_{1.9 \times 10^{-3}}$	$5.67 \times 10^{-1}_{1.6 \times 10^{-3}}$	$5.39 \times 10^{-1}_{2.5 \times 10^{-2}}$
4zau	$5.95 \times 10^{-1}_{5.5 \times 10^{-3}}$	$6.70 \times 10^{-1}_{4.3 \times 10^{-2}}$	$6.85 \times 10^{-1}_{7.4 \times 10^{-2}}$	$6.70 \times 10^{-1}_{5.9 \times 10^{-1}}$
G719S / L858R				
2j6m	$8.60 \times 10^{-1}_{5.8 \times 10^{-3}}$	$8.84 \times 10^{-1}_{4.4 \times 10^{-3}}$	$8.69 \times 10^{-1}_{6.6 \times 10^{-3}}$	$8.82 \times 10^{-1}_{1.4 \times 10^{-2}}$
T790M / G719S				
3ug2	$4.13 \times 10^{-1}_{2.1 \times 10^{-2}}$	$4.42 \times 10^{-1}_{2.5 \times 10^{-2}}$	$4.57 \times 10^{-1}_{2.1 \times 10^{-2}}$	$4.36 \times 10^{-1}_{2.5 \times 10^{-2}}$
3vjn	$0.00 \times 10^{+0}_{0.0 \times 10^{+0}}$	$0.00 \times 10^{+0}_{0.0 \times 10^{+0}}$	$6.04 \times 10^{-1}_{1.9 \times 10^{-1}}$	$0.00 \times 10^{+0}_{2.7 \times 10^{-1}}$
G719S				
2itn	$7.48 \times 10^{-1}_{2.0 \times 10^{-2}}$	$7.72 \times 10^{-1}_{4.5 \times 10^{-2}}$	$7.75 \times 10^{-1}_{3.4 \times 10^{-2}}$	$7.25 \times 10^{-1}_{1.1 \times 10^{-1}}$
2ito	$4.35 \times 10^{-1}_{2.0 \times 10^{-2}}$	$4.70 \times 10^{-1}_{6.1 \times 10^{-2}}$	$5.04 \times 10^{-1}_{6.1 \times 10^{-2}}$	$5.11 \times 10^{-1}_{6.2 \times 10^{-2}}$
2itp	$5.57 \times 10^{-1}_{1.2 \times 10^{-2}}$	$6.09 \times 10^{-1}_{7.0 \times 10^{-3}}$	$6.05 \times 10^{-1}_{5.8 \times 10^{-2}}$	$5.34 \times 10^{-1}_{3.8 \times 10^{-1}}$
2itq	$6.05 \times 10^{-1}_{1.1 \times 10^{-2}}$	$6.45 \times 10^{-1}_{3.1 \times 10^{-3}}$	$6.49 \times 10^{-1}_{7.0 \times 10^{-3}}$	$6.38 \times 10^{-1}_{9.8 \times 10^{-3}}$
L858R				
2eb3	$5.58 \times 10^{-1}_{1.1 \times 10^{-1}}$	$6.68 \times 10^{-1}_{8.8 \times 10^{-2}}$	$6.88 \times 10^{-1}_{9.8 \times 10^{-2}}$	$6.40 \times 10^{-1}_{8.9 \times 10^{-2}}$
2itu	$5.75 \times 10^{-1}_{2.3 \times 10^{-2}}$	$5.29 \times 10^{-1}_{1.1 \times 10^{-3}}$	$6.05 \times 10^{-1}_{8.6 \times 10^{-3}}$	$5.33 \times 10^{-1}_{6.2 \times 10^{-2}}$
2itz	$4.11 \times 10^{-1}_{1.6 \times 10^{-2}}$	$5.40 \times 10^{-1}_{3.3 \times 10^{-2}}$	$5.09 \times 10^{-1}_{5.9 \times 10^{-2}}$	$4.90 \times 10^{-1}_{8.1 \times 10^{-2}}$
T790M				
2jiu	$4.99 \times 10^{-1}_{3.3 \times 10^{-3}}$	$5.13 \times 10^{-1}_{1.0 \times 10^{-2}}$	$5.25 \times 10^{-1}_{8.6 \times 10^{-3}}$	$5.12 \times 10^{-1}_{1.5 \times 10^{-2}}$
5j9y	$8.18 \times 10^{-1}_{1.4 \times 10^{-3}}$	$8.22 \times 10^{-1}_{2.2 \times 10^{-4}}$	$8.78 \times 10^{-1}_{1.4 \times 10^{-2}}$	$8.22 \times 10^{-1}_{2.7 \times 10^{-1}}$
5j9z	$9.01 \times 10^{-1}_{8.6 \times 10^{-4}}$	$9.05 \times 10^{-1}_{4.4 \times 10^{-5}}$	$9.23 \times 10^{-1}_{3.8 \times 10^{-3}}$	$9.05 \times 10^{-1}_{1.6 \times 10^{-4}}$
T790M / L858R				
3w2s	$5.65 \times 10^{-1}_{2.4 \times 10^{-2}}$	$0.00 \times 10^{+0}_{0.0 \times 10^{+0}}$	$6.31 \times 10^{-1}_{2.3 \times 10^{-2}}$	$0.00 \times 10^{+0}_{0.0 \times 10^{+0}}$
4rj4	$4.70 \times 10^{-1}_{8.7 \times 10^{-2}}$	$6.89 \times 10^{-1}_{3.3 \times 10^{-2}}$	$8.71 \times 10^{-1}_{6.7 \times 10^{-2}}$	$6.19 \times 10^{-1}_{4.3 \times 10^{-2}}$
4rj5	$2.07 \times 10^{-1}_{1.9 \times 10^{-2}}$	$2.11 \times 10^{-1}_{3.8 \times 10^{-3}}$	$2.16 \times 10^{-1}_{2.0 \times 10^{-2}}$	$2.20 \times 10^{-1}_{4.1 \times 10^{-2}}$
4rj6	$2.48 \times 10^{-1}_{5.8 \times 10^{-2}}$	$4.90 \times 10^{-1}_{1.0 \times 10^{-2}}$	$8.55 \times 10^{-1}_{5.9 \times 10^{-2}}$	$4.60 \times 10^{-1}_{4.8 \times 10^{-2}}$
4rj7	$6.64 \times 10^{-1}_{7.6 \times 10^{-3}}$	$7.08 \times 10^{-1}_{2.6 \times 10^{-3}}$	$7.13 \times 10^{-1}_{2.0 \times 10^{-2}}$	$5.40 \times 10^{-1}_{6.9 \times 10^{-1}}$
4rj8	$6.16 \times 10^{-1}_{1.1 \times 10^{-2}}$	$6.44 \times 10^{-1}_{6.0 \times 10^{-3}}$	$6.47 \times 10^{-1}_{7.8 \times 10^{-3}}$	$6.11 \times 10^{-1}_{2.2 \times 10^{-2}}$
5c8k	$5.92 \times 10^{-1}_{1.0 \times 10^{-2}}$	$6.15 \times 10^{-1}_{1.2 \times 10^{-3}}$	$6.20 \times 10^{-1}_{9.7 \times 10^{-3}}$	$5.34 \times 10^{-1}_{2.6 \times 10^{-2}}$
5c8m	$5.49 \times 10^{-1}_{5.5 \times 10^{-3}}$	$5.74 \times 10^{-1}_{5.5 \times 10^{-3}}$	$5.77 \times 10^{-1}_{1.9 \times 10^{-3}}$	$5.68 \times 10^{-1}_{2.0 \times 10^{-1}}$
5cal	$5.70 \times 10^{-1}_{2.0 \times 10^{-2}}$	$5.95 \times 10^{-1}_{6.4 \times 10^{-3}}$	$6.34 \times 10^{-1}_{4.7 \times 10^{-2}}$	$5.96 \times 10^{-1}_{6.5 \times 10^{-1}}$
5can	$5.52 \times 10^{-1}_{2.8 \times 10^{-2}}$	$5.58 \times 10^{-1}_{4.7 \times 10^{-3}}$	$6.15 \times 10^{-1}_{5.5 \times 10^{-2}}$	$5.44 \times 10^{-1}_{1.4 \times 10^{-1}}$
5cao	$4.66 \times 10^{-1}_{1.3 \times 10^{-2}}$	$0.00 \times 10^{+0}_{2.7 \times 10^{-1}}$	$5.48 \times 10^{-1}_{5.4 \times 10^{-2}}$	$2.37 \times 10^{-1}_{4.6 \times 10^{-1}}$
5cap	$4.37 \times 10^{-1}_{8.1 \times 10^{-2}}$	$5.61 \times 10^{-1}_{2.1 \times 10^{-1}}$	$5.66 \times 10^{-1}_{3.8 \times 10^{-2}}$	$3.81 \times 10^{-1}_{2.0 \times 10^{-1}}$
5caq	$6.53 \times 10^{-1}_{6.2 \times 10^{-3}}$	$6.75 \times 10^{-1}_{6.6 \times 10^{-3}}$	$6.80 \times 10^{-1}_{1.1 \times 10^{-2}}$	$5.96 \times 10^{-1}_{6.7 \times 10^{-2}}$
5cas	$6.07 \times 10^{-1}_{1.3 \times 10^{-2}}$	$6.31 \times 10^{-1}_{3.7 \times 10^{-3}}$	$6.26 \times 10^{-1}_{9.4 \times 10^{-3}}$	$5.89 \times 10^{-1}_{3.0 \times 10^{-2}}$
5cau	$5.96 \times 10^{-1}_{2.3 \times 10^{-2}}$	$6.20 \times 10^{-1}_{2.2 \times 10^{-3}}$	$6.20 \times 10^{-1}_{1.2 \times 10^{-2}}$	$5.49 \times 10^{-1}_{7.8 \times 10^{-2}}$
5em5	$5.95 \times 10^{-1}_{9.8 \times 10^{-2}}$	$5.75 \times 10^{-1}_{3.5 \times 10^{-1}}$	$6.13 \times 10^{-1}_{3.8 \times 10^{-1}}$	$4.82 \times 10^{-2}_{3.5 \times 10^{-1}}$
5em7	$7.37 \times 10^{-1}_{2.9 \times 10^{-2}}$	$8.23 \times 10^{-1}_{8.1 \times 10^{-3}}$	$7.79 \times 10^{-1}_{4.9 \times 10^{-2}}$	$7.62 \times 10^{-1}_{5.7 \times 10^{-2}}$
5em8	$4.37 \times 10^{-1}_{6.5 \times 10^{-2}}$	$5.00 \times 10^{-1}_{9.0 \times 10^{-3}}$	$5.22 \times 10^{-1}_{1.5 \times 10^{-1}}$	$5.15 \times 10^{-1}_{9.9 \times 10^{-2}}$
5hic	$5.58 \times 10^{-1}_{1.9 \times 10^{-2}}$	$0.00 \times 10^{+0}_{6.1 \times 10^{-1}}$	$6.07 \times 10^{-1}_{4.3 \times 10^{-3}}$	$5.92 \times 10^{-1}_{6.0 \times 10^{-1}}$
